# Vigo on Toothache, 1514

**Published:** 1899-01

**Authors:** 


ARTICLE IV.

### VIGO ON TOOTHACHE, 1514.

Regarding the general anatomy of the teeth and the surrounding parts, this writer had a fairly accurate knowledge, but of their minute anatomy he knew absolutely nothing. He begins the chapter on toothache by saying: "The teethe are wont to be vexed through a reumatyke matter distillinge fro ye brayne, and through the fauet of the stomake, when under sondrye passions, but seynge that the teethe serve for comelynes, for chewynge of meate and for pronunciation, therefore they must be cured wyth all diligence." Quoting from an earlier writer, he names six diseases of the teeth, pain, corrosion, congelation, dormitation, filthiness and looseness." Pain seems not to have been considered a symptom of a disordered condition, but the disorder itself; and a vague idea was entertained that this pain was in some way due to an inflammation of the tooth substance, and was more severe than other pains, because on account of the hardness of the teeth this inflammation was not relieved by suppuration as in the soft tissues. He states, still quoting from older writers, "The teethe have no feeling in themselves, but by reason of nerves which come from the third pair of nerves of the brain to their roots and to the gums; this is evident, for when one little piece of the tooth is broken the man is not pained. The pain comes by an evil complexion of the nerve, or a hot or cold aposteme (abscess), wherefore, sometimes the pain is aswaged when the tooth is pulled up, and the matter which caused the pain is allowed to escape." This manoeuvre, it is added, also permits the medicine to enter more easily and ease the painful place. When all else fails, on the authority of Galen, we are advised to pull the tooth out by the roots.

Corrosion, we readily recognize as caries; and he recommends that the corrosion be removed with trephines, files and other convenient instruments, the cavity to be then filled with leaves of gold to preserve the place from putrefaction.

Congelation, he tells us, "chanceth to the teeth of outward or inward things. Of outward, when a man eateth sour things, of inward, when lower vapors ascend from the stomach. Also there is yellow filth sticking to the teeth and the roots thereof, which comes of gross vapors ascending from the stomach, and which may be removed by rubbing with convenient instruments." He recommends the following powder for cleansing the teeth, preserving the gums, making the teeth white, and remedying the stinking of the mouth: "℞ Rock Alum, burnt, 2 dr.; sarcocolla (Persian gum), terra figillate (potter's clay), mitahola citrine (lemon peel), of each 1 dr., mingle them together, and make a powder and rub the teeth therewith, in the morning fasting, twice a week."

Dormitation is a disease of the teeth referred to by the old writers which I am unable to recognize. This writer says it is caused by holding cold things, or stupefying medicines in the mouth, and recommends for its removal the following, to be held in the mouth warm.

℞ Odoriferous wine, half a pound; aqua vita, 1 oz.; rosemary, sage, and camomile, of each a scruple; sandrake, 1 dr. Boil these together until one third part is consumed, then strain them, and use as directed. Aqua vita, applied to the teeth with cotton, takes away the dormitation and congelation." Both these expressions originated with the ancient writers, and are found repeated in medico-dental works like this. In none have I so far found a description sufficiently exact to identify them with conditions met with in practice.

Looseness of the teeth, he tells us, arises from a looseness of the gums, or through debility or weakness of the roots, or of the parts which bind the teeth, caused by rheums and humors descending from the brain, sometimes by corrupt vapors mounting from the stomach. For the cure of this disorder he recommends the following medicine : "℞ Syrup liciti, 10 dr.; rock alum, 3 dr.; water of plantain, 1½ dr.; wine of pomegranate, 3 dr.; honey of rose, 6 dr.; Persian gum, ½ dr.; vinegar of squills ½ oz.; leaves of wild olives somewhat bruised, half a handful. These, except the liciti and honey of rose, are to be sodden together, then strained, then sethed again with the liciti and honey of rose until two parts out of three are consumed." The gums are to be rubbed with this composition, "for it fastens the teeth, and removes the putrefaction, and comforts the nerves which come to the roots of the teeth." If a more desicative medicine be required, he recommends the unguentum Egypticum of Avicenne, "which hath virtue to remove the evil and to conserve the good." We have in these two medicines the combination of astringent and sedative, the last partaking somewhat of a caustic. We would to-day, in a like condition, with better prepared and more active agents, still use astringent and sedative remedies, resorting to caustics if more potent means were required. He then goes on to say : "Besides the six causes before named, pain of the teeth may come of worms, which are engendered in the hollowness of the same, or by abscesses of the gums."

He next speaks of "hot and cold apostemes." I gather from him and other writers of that period, that by these expressions they referred to abscesses accompanied by inflammation, or periosteal irritation, and others where the inflammation was not so pronounced; and in this connection they also included exposed pulps. The science, as expounded by medical writers, had not yet reached the point when these several conclusions were recognized as separate and distinct. A hot pain was one which was eased by cold applications, a cold pain one which ceased on hot applications being made. For the relief of these conditions resort was had to hot or cold applications, to purging, to local medication, and to diet. In addition, cleanliness of the mouth and teeth is urged, and that the patient's wine should be of good order, and mingled with warm water. On the authority of Mesue, as a farther measure of relief, if the matter is hot, blood letting, a blister on the shoulder or nape of the neck, scarrification of the gums and application of blood-suckers (leeches), are recommended. Nearly all the remedies are more or less astringent in character. Vinegar was a stand by, in dental medicine, from its supposed ability to penetrate the the hard tissues of the teeth, and carry to the seat of pain the virtues of the various remedies combined with it. A favorite remedy ascribed to Avercenne, was vinegar in which an adder's skin had been steeped, or a decoction of a frog sodden in vinegar and water. Mesue recommends camphor, corriander seed, the juice of nightshade, and stomach, in tbe relief of tooth pain. At the time this book was written, the medical profession was in close bondage to the old masters, so far as its text books were concerned. The light was beginning to break, but personal experience was apologetically expressed, and opposed very cautiously indeed to the accepted tradition of the past.

He gives a number of remedies for real or fancied diseases of the teeth; most of them are vegetable decoctions and stews, containing a large number of ingredients, which we of today consider of but little value. Gum camphor, alum, acetic acid, alcohol, and a few aromatics, are all that have retained their place in modern dental materia medica. He closes the chapter with a plea for more earnest attention to disorders of the teeth, gums, and associate parts; and suggests that those surgeons who are so ready to recommend their patient to the barkers and wandering vagabond tooth drawers, would do well to see these worthies at their work. Whether this remark is intended to be a reflection upon the surgeons, or the tooth drawers, is not clear.—*Items of Interest.*

